# General Precautions in the Administration of Anæsthetics

**Published:** 1885-02

**Authors:** 


### General Precautions in the Administration of Anæsthetics.

While we are frequently taught that we must be very careful in the administration of anæsthetics, yet it is well that this caution should be constantly impressed upon our memories, hence we deem it well to reproduce the conclusions with which Dr. George Eastes concludes his paper in the *Brit. Med. Jour.*, November 29, 1884. They are as follows:

1. The patient should have no meal (except in cases of extreme feebleness) for four hours beforehand, to avoid the tendency to vomiting. A little brandy or ammonia may be given with some water fifteen minutes before the operation if the patient be an adult and chloroform be selected.

2. The anæsthetist should endeavor to reassure the patient by a kind, gentle manner, which may calm an agitated heart, and induce easy respiration.

3. No tight-fitting garment or band should be left around the chest or abdomen.

4. All artificial teeth must be removed from the mouth.

5. The anæsthetist should examine the heart-sounds and pulse before the operation. This precaution may seem superfluous, in most instances, but in a few cases may put the administrator on extra guard.

6. The patient should be placed in the recumbent posture, with the head but slightly raised. This is particularly important when chloroform or any other cardiac depressor is used.

7. The anæsthetic should be applied in the manner most suitable to the vapor used, about which I have already sufficiently spoken under the heading of each drug that I have mentioned.

8. The surgeon should not commence his procedure before the patient is fully narcotized; not alone in severe operations, for many simple procedures, such as the reduction of dislocated joints, and strangulated hernia, and the diagnosing of tumors of the abdomen and pelvis, require complete muscular relaxation for their successful attainment.

9. It is a golden rule never to give more of the anæsthetic than is necessary for the production of sufficient anæsthesia for the operation that has to be performed. Since every extra degree in anæsthesia is an advance along that road whose extreme goal is death, the patient should be taken no nearer the dreaded end than is quite requisite, as all the steps passed have to be retraced. Besides, as the gap separating the patient from death becomes lessened, it may easily arise that, in extreme narcosis, one extra and apparently trivial event, which in health would be quite inoperative to cause a fatal issue, may determine the patient's career.

10. Wherefore, lastly, as the work which the anæsthetist performs depends for its successful result on such a number of nicely-balanced conditions, his whole attention, during the few minutes of his employment, should be concentrated upon the case. He must watch the respirations, the pulse, the countenance, and the general condition of the patient. He should not be observing attentively the steps of the surgeon's procedure, otherwise he may fail to notice the first sign of danger in the patient, and the life, for which he is chiefly responsible, may be jeopardized before it is recognized that it is in peril. Moreover, the anæsthetist may rely upon receiving more than full recompense for all his care in a decreased number of mischances and a welcome table of good results.

